# A long-term follow-up of a girl with dilated cardiomyopathy after mitral valve replacement and septal anterior ventricular exclusion

**DOI:** 10.1186/1749-8090-4-53

**Published:** 2009-09-23

**Authors:** Shiro Baba, Hiraku Doi, Tadashi Ikeda, Masashi Komeda, Tatsutoshi Nakahata

**Affiliations:** 1 Department of Pediatrics, Graduate School of Medicine, Kyoto University, 54 Kawahara-cho, Shogoin, Sakyo-ku, Kyoto 606-8507, Japan; 2 Department of Cardiovascular Surgery, Graduate School of Medicine, Kyoto University, 54 Kawahara-cho, Shogoin, Sakyo-ku, Kyoto 606-8507, Japan; 3 Department of Cardiovascular Surgery, Toyohashi Heart Center, 1-2 Gobudori, Ohyama-cho, Toyohashi, Aichi 441-8530, Japan

## Abstract

We treated a 10 year 11 month old girl with severe mitral valve regurgitation, stenosis and dilated cardiomyopathy, presented with New York Heart Association (NYHA) functional classification IV. She acutely developed cardiogenic shock with a dyskinetic anterior-septal left ventricle and entered a shock state during our consultation about heart transplantation. Septal-anterior ventricular exclusion and mitral valve replacement were performed emergently. She successfully recovered from cardiogenic shock. Left ventricular end-diastolic diameter and fractional shortening improved from 71.5 mm (188.0% of normal) to 62.5 mm (144.2% of normal) and 7.6% to 18.3% respectively. Furthermore, her serum BNP decreased from 2217.5 pg/ml to 112.0 pg/ml. Her cardiac function has remained stable for 7 years since the procedures were performed.

## Background

Dilated cardiomyopathy (DCM) is one of the most serious prognostic factors in heart disease [1,2]. Batista et al. described left ventriculectomy in 1996 which has become one of the most important surgical therapies for adults with DCM [3-6]. However, in patients with both damaged intraventricular septum (IVS) and damaged left ventricular (LV) free wall, cardiac function worsens following this procedure. The Dor procedure and Septal Anterior Ventricular Exclusion (SAVE) procedures have recently been recommended in these patients [7-9].

## A Case Presentation

In November 2001, a 10 year, 11 month old girl was admitted to our hospital with dyspnea on mild exertion and pretibial and palpebral edema.

At 2 months, a heart murmur was detected. One year later, she was diagnosed with congenital mitral valve stenosis (MS) and mitral valve regurgitation (MR) by cardiac echogram and catheterization. Despite treatment with digitoxin and diuretics, her left ventricular end-diastolic diameter (LVDd) gradually increased and MR worsened. She received mitral valve replacement (MVR) at age 6, but her cardiac function continued to worsen and her LVDd increased despite of 9 years optimal medical treatment.

**Figure 1:**
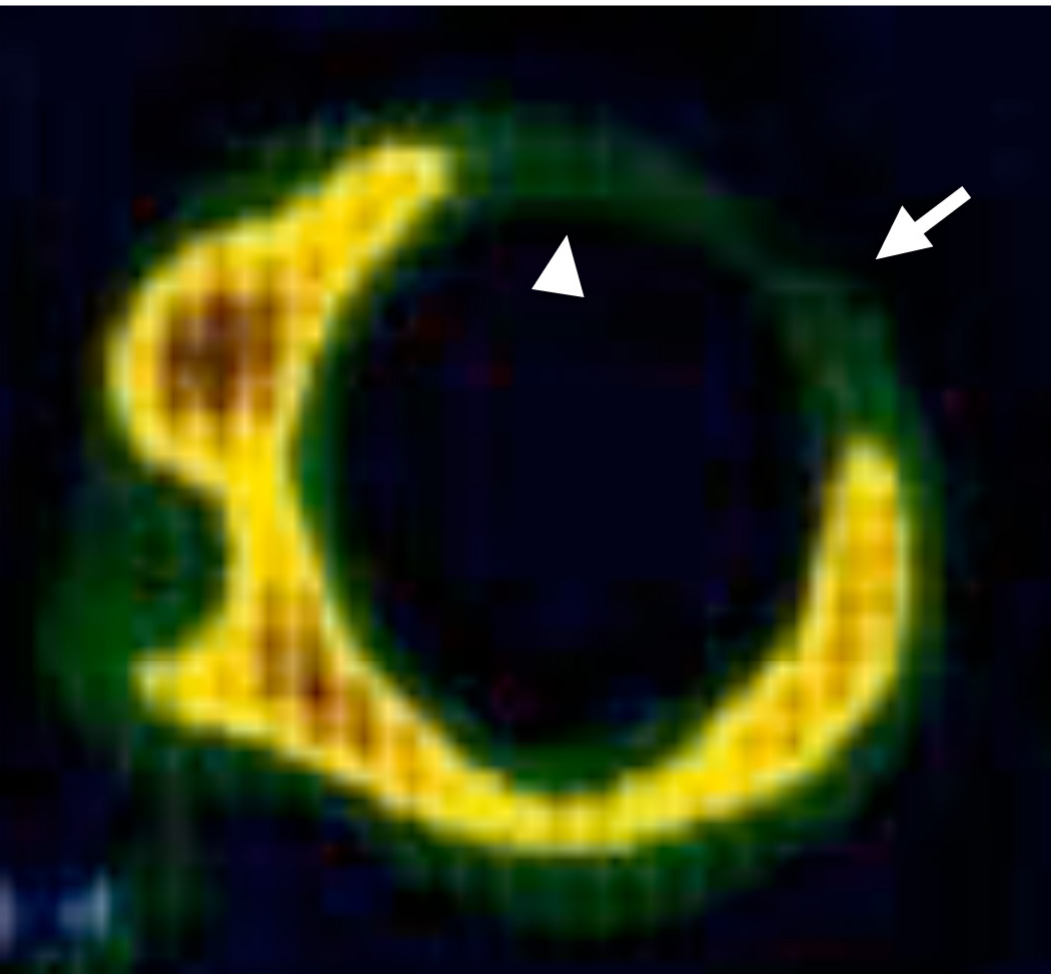
**^201^Tl uptake was decreased from the anterior part of the IVS and anterior wall of the LV on cardiac scintigraphy**. (Arrow: Anterior wall of LV, Arrowhead: Anterior part of IVS).

**Figure 2:**
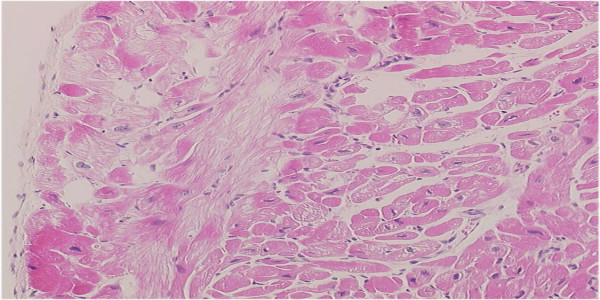
**Fibrotic change and vacuolar degeneration in the excised IVS specimen**.

At the time of her hospitalization, a chest X-ray revealed pulmonary congestion and cardiomegaly (cardio-thoracic ratio 79.0%). Echocardiogram showed dilated LVDd, of 71.5 mm (188% of normal), reduced left ventricular fractional shortening (LVFS) (7.6%) and closure of one of the artificial mechanical valves. Left ventricular ejection fraction (LVEF) was also measured by cardiac catheterization, and the LVEF was 11.0% at this time. Serum BNP was elevated at 2217.5 pg/ml. Decreased up-takes of ^201^Tl and ^123^I-MIBG were detected in the anterior IVS and anterior LV wall by cardiac scintigraphy (Figure 1). A cardiac muscle biopsy revealed fibrous and vacuolar degeneration in the IVS area (Figure 2). Both the left and right coronary arteries were normal and there was no evidence of ischemic cardiomyopathy by an angiogram.

Despite of treatment with bed-rest, diuretics and cardiotonic agents, her condition continued to worsen. While preparing to place her on the heart transplant waiting list, she went into a cardiogenic shock requiring mechanical ventilation and placement of an intra-aortic balloon pumping (IABP). Soon after the onset of the shock, SAVE procedure and the second MVR were performed emergently. We replaced a 23 mm diameter St. Jude Medical mechanical valve and tied up and patched the thin area of her anterior IVS and anterior LV wall with a sheet of patch after a close examination of her LV wall by intra-operative echocardiogram. Her LVDd decreased to 52.8 mm (139.0% of normal) after 1 and 62.5 mm (144.2% of normal) after 7 years of the SAVE procedure. Her LVFS elevated to 15.4% after 1 and 18.3% after 7 years of the SAVE procedure. Serum BNP remarkably decreased to 129.3 pg/ml after 1 and 112.0 pg/ml after 7 years of the SAVE procedure. Upon cardiac catheterization, LVEF had increased and LV volume index had not changed between 2 months after (16.6% and 180.6 ml/m^2^, respectively) and 7 years after (36.5% and 173.7 ml/m^2^, respectively) the SAVE procedure. Although single and monofocal premature ventricular conductions are occasionaly recorded on electrocardiography, her condition is stable and she is able to attend high school daily by wheelchair.

## Conclusion

Severe heart failure in children is commonly treated with diuretics, ACE inhibitors, calcium blockers, β-blockers and vasodilators [10,11]. Patients with DCM and NYHA functional class, who do not respond to medical therapy, are candidates for heart transplantation. In addition to the shortage of available organs, there are legal, economical, ethical and technical problems associated with heart transplantation in many countries.

Randas Batista et al. described techniques to improve cardiac contraction and reduce LV diameter [3]. But damage may extend beyond the LV free wall. The Dor and SAVE procedures have improved outcomes for patients with damaged IVS [7-9]. These procedures recommend resection or exclusion of both the non-functioning parts of the IVS and the LV fee wall. Since the non-functional wall is not removed but excluded with a patch in the SAVE procedure, the SAVE procedure is better in cardiac function improvement, particularly for the patients with large areas of damaged IVS such as our patient.

LV diameter reduction has been performed worldwide in adults and has been shown to improve LV function in mid-term follow-up studies. However, long-term follow-up after the SAVE procedure [12], especially in children, has been limited. 7 years after the SAVE procedure, our patient is doing well, enjoying daily life requiring little assistance. Many children with severe heart failure cannot receive transplantation quickly because of numerous problems. We recommend the SAVE procedure not only for adults, but also for children with a large non-functional LV area. Although we can not conclude that this SAVE procedure is an equally efficacious alternate to heart transplantation, the procedure appears to be at least a bridging treatment for use between medical treatment and heart transplantation [9].

In conclusion, we report good long-term outcome in a child with DCM and large non-functional LV area treated with the SAVE procedure. She recovered from cardiogenic shock and her cardiac function has now been stable for more than 7 years after the SAVE procedure.

## Abbreviations

NYHA: New York Heart Association; DCM: dilated cardiomyopathy; IVS: intraventricular septum; LV: left ventricular or left ventricle; SAVE: Septal Anterior Ventricular Exclusion; MS: mitral valve stenosis; MR: mitral valve regurgitation; LVDd: left ventricular end-diastolic diameter; MVR: mitral valve replacement; LVFS: left ventricular fractional shortening; IABP: intra-aortic balloon pumping.

## Competing interests

The authors declare that they have no competing interests.

## Authors' contributions

SB was an attending physician in the pediatric ward in Kyoto university hospital, and wrote most part of this manuscript. HD is an attending physician in the pediatric outpatient clinic in Kyoto university hospital, and gave some comments for this manuscript. TI was an assistant operator in the SAVE operation. MK is a chief operator in the SAVE operation. TN is a general supervisor of this manuscript.

## Authors' Informations

SB is an assistant professor and a pediatric cardiologist in charge in a pediatric ward of Kyoto university hospital. HD is an assistant professor and a pediatric cardiologist in charge in a pediatric ward and an outpatient clinic of Kyoto university hospital. TI is an associate professor in the department of cardiovascular surgery in Kyoto university hospital. MK is a previous professor of the department of cardiovascular surgery in Koyto university hospital. Now he works as a cardiovascular surgeon in Toyohashi heart center. TN is a professor of the pediatrics department in Kyoto university hospital. He is a supervisor of this manuscript.

## Consent

Written informed consent was obtained from this patient and her mother for publication of this case report and any accompanying images. A copy of the written consent is available for review by the Editor-in-Chief of this journal.
